# In Vitro fertilization failure of normozoospermic men: search for a lack of testicular isozyme of angiotensin-converting enzyme

**DOI:** 10.1186/2051-4190-23-4

**Published:** 2013-08-29

**Authors:** Selima Fourati Ben Mustapha, Florence Coulet, Mélanie Eyries, Vanina De Larouziere, Celia Ravel, Isabelle Berthaut, Jean-Marie Antoine, Florent Soubrier, Jacqueline Mandelbaum

**Affiliations:** Clinique de Promotion des Sciences de la Reproduction (CPSR) Les Jasmins, 23, Avenue Louis Braille, 1002 Tunis, Tunisia; INSERM U525, UPMC6, Pitié-Salpétrière site, Paris, France; Department of Histology and Biology of Reproduction, Tenon Hospital (Assistance Publique Hôpitaux de Paris), UPMC, Paris 6, France; Department of obstetrics and gynecology, Tenon Hospital (Assistance Publique Hôpitaux de Paris), Paris, France

**Keywords:** Angiotensin converting enzyme, Male infertility, IVF failure, Spermatozoa, Zona pellucida, Sperm-oocyte interaction, Enzyme de conversion de l′angiotensine, Infertilité masculine, Echec de FIV, Spermatozoïde, Zone pellucide, Interaction spermatozoïde-ovocyte

## Abstract

**Background:**

Angiotensin converting enzyme (ACE) is a metalloprotease with two isoforms. The somatic isoform is a key component of the renin-angiotensin system; its main function is to hydrolyse angiotensin I into angiotensin II. The germinal or testicular isoform (tACE) located at the plasma membrane of the spermatozoa, plays a crucial role in the spermatozoa-oocyte interaction during *in vivo* fertilization, in rodents. Disruption of the tACE in mice has revealed that homozygous male tACE−/− sire few pups despite mating normally. Few spermatozoa from these tACE−/− mice are bound to the zona pellucida (ZP) despite normal semen parameters. Based on these findings in mice models, we hypothesized that some infertile men that have the same phenotype as the tACE−/− mice, ie normal semen parameters and a lack of sperm bind to the ZP *in vitro*, may have a tACE defect.

**Methods:**

Twenty four men participated to this study. The case subjects (n = 10) had normal semen parameters according to the WHO guidelines (WHO 1999) but a total *in vitro* fertilization failure with absence of sperm fixation to the ZP. The control subjects (n = 14) also had normal semen parameters and a normal fertilization rate ≥65%. We investigated the tACE expression in spermatozoa by Western-Blot and performed a DNA sequencing of the tACE gene.

**Results:**

Three case-subjects and one control-subject had no tACE expression. There were no statistic differences between the two groups. No mutation was detected in the tACE DNA sequence.

**Conclusions:**

Our results didn’t show any involvement of tACE in human fertilization especially in ZP binding.

## Résumé

**Introduction:**

L’enzyme de conversion de l′angiotensine (ACE) est une métalloprotéase qui se présente sous deux isoformes. Une isoforme somatique qui intervient dans la régulation de la pression artérielle via le système rénine-angiotensine et une isoforme testiculaire spermatique (ACEt) qui interviendrait dans la fixation du spermatozoïde aux enveloppes ovocytaires (zone pellucide et/ou oelemme). Des expériences d’invalidation du gène de l′ACEt chez la souris ont montré que les mâles homozygotes ACEt−/− présentaient une faible descendance *in vivo* et une fixation faible des spermatozoïdes à la zone pellucide (ZP) *in vitro* et ce malgré un sperme normal. Ces résultats nous ont permis d’émettre l′hypothèse que certains échecs de fécondation en fécondation *in vitro* classique (FIV) chez des patients à sperme normal seraient en rapport avec un déficit de l′ACEt au niveau des spermatozoïdes.

**Matériel et Méthodes:**

Vingt quatre patients ont été inclus dont 10 cas avec un sperme normal et un échec total de fécondation lors d’une FIV et 14 témoins ayant les mêmes caractéristiques spermatiques mais présentant de bons taux de fécondation (≥65%). L’étude de l’expression protéique de l’ACEt a été faite par western-blot sur les culots de spermatozoïdes. Un séquençage du gène codant pour l′ACEt a été fait sur l′ADN lymphocytaire.

**Résultats:**

Trois cas et un témoin présentaient un défaut d’expression de l′ACEt sans diffèrence statistiquement significative entre ces deux groupes. En PCR, aucune mutation n’a été retrouvée après séquençage du gène de l′ACEt.

**Conclusions:**

Nos résultats n’ont pas objectivé une implication de l′ACEt dans l′interaction entre ovocyte et spermatozoïde et notamment dans la fixation à la ZP.

## Introduction

In standard *in vitro* fertilization (IVF), 5 to 10% of patients have a complete failure of fertilization [1]. It may result from impaired spermatozoa, oocyte deficiencies or defects in the *in vitro* sperm-oocyte interaction. In assisted reproductive technology programs, 70% of mature oocytes will successfully fertilize. Thus, each oocyte has 30% of chance of fertilization failure and the likelihood of failed fertilization would depend on the number of available oocytes (n) and is (0.3)^n^. Thus, the chance of failed fertilization in a couple with three mature oocytes should be approximately 2.7% [1] and in these cases, sperm defects appear to be the major cause although semen parameters are normal. Strongly supporting this hypothesis is the observation that failed fertilized oocytes reincubated with donor spermatozoa usually fertilize [1]. This sperm defect can lead either to a total or a partial absence of sperm binding to the ZP [1, 2] or the absence of acrosome reaction [2, 3], or the defect of spermatozoon access to the perivitelline space and his attachment to the oelemma membrane.

Angiotensin converting enzyme (ACE) is a Zn^2+^ metalloprotease dipeptidyl carboxypeptidase widely distributed in mammalian organisms. It was first identified as a key component of the renin-angiotensin system (RAS) where its main function was to hydrolyse the inactive peptide, angiotensin I, into the physiologically active octapeptide angiotensin II. Angiotensin II plays an important role in controlling fluid electrolyte balance and systemic blood pressure [4–6]. ACE also inactivates the vasodilator peptide bradykinin by removal of its two coterminal dipeptides [7].

Two isoforms of ACE are known to exist in mammals. The larger protein, the somatic ACE (sACE) is expressed widely in the body. It is found in blood, vascular endothelial cells, epithelial cells of the proximal tubules of the kidney, brain, intestinal brush border [8, 9]. Epididymis, macrophages and testis Leydig cells also express this isozyme [10].

In contrast, the testicular or germinal isoform (tACE) is exclusively found in male germinal cells. In murine testis, tACE is found to be expressed in post-meiotic germinal cells during spermiogenesis [11] whereas in human testis, this protein is found in the golgi apparatus of spermatids at different stages of spermiogenesis. In ejaculated human spermatozoa, immunoelectron microscopy demonstrated that tACE is mainly located at the plasma membrane of the acrosomal region, equatorial segment, post acrosomal region and mid piece [12].

Both somatic and testicular ACE are encoded by the same gene consisting of 26 exons on chromosome 17q23 via two different tissue-specific promoters. Somatic ACE is transcribed from a promoter region upstream exon 1 in the 5′ region and is composed of a single polypeptide chain with two homologous domains, each one bearing an active catalytic site [7]. In contrast, tACE is transcribed from a promoter within intron 12 and it has only the active carboxyterminal domain of the sACE [13, 14].

Disruption of the mouse ACE gene has revealed the multiple physiological roles of ACE. Homozygous ACE−/− mice had lower blood pressure, atrophy of the renal cortex, thickened arteries and are unable to concentrate urine or maintain normal urinary salt balance. Besides, male ACE−/− mice sire few pups although they have normal testis structure and semen parameters (count, morphology and motility) [8, 15].

Transgenic mice lacking the somatic isoform but not the testicular one have the same renal and vascular abnormalities as those above. However, males’ sACE−/− are fertile [9, 16]. Moreover, angiotensinogen gene knockout male mice are fertile suggesting that this molecule is not the substrate of the tACE [8].

Transgenic male mice that express only the sACE but not the testicular one in the testis sire few pups despite mating normally [17].

The decreased fertility of these tACE−/− mice can be fully accounted for by the reduced capacity of their sperm to achieve fertilization *in vivo* as demonstrated by Hagaman et al. [16]. Indeed, despite normal functional parameters, very few spermatozoa from tACE−/− male mice are found in oviduct female regions 1 hr after mating compared to the wild mice. Thus, less than 5% of the eggs were fertilized *in vivo* from these tACE−/− mice matings versus 65% of the eggs from the wild mice matings [16]. The fertilized eggs by sperm from tACE−/− mice develop normally to the 8-cell stage *in vitro* and these males sire occasional pups [16]. Besides, in an *in vitro* assay, sperm from tACE−/− mice show defects in binding to ZP [16]. Based on these findings in mice models, we hypothesized that some infertile men that have the same phenotype as the tACE−/− mice, i.e. normal semen parameters (number, motility and morphology) and a defect of their spermatozoa to bind to the ZP in an *in vitro* fertilization cycle, may have a tACE defect. First, we investigated the tACE expression in spermatozoa, and then we performed a DNA sequencing of the tACE gene.

## Material and methods

From January 2004 until December 2005 patients were included in this prospective study at the department of Histology and Biology of Reproduction at Tenon Hospital.

### Patient selection

Twenty-four men participated to this study. All of them were in good physical and mental health. All the men (cases and controls) have a primary infertility and are members of couples undergoing their first *in vitro* fertilization cycle. Patients with genital tract infection, congenital disease or treated with ACE inhibitors were excluded from this study.

The case subjects (n = 10) have normal semen parameters (number, motility, morphology) according to the WHO guidelines [18] but a total *in vitro* fertilization failure occurring during an IVF cycle. The causes of infertility were tubal infertility (4 cases) and unexplained infertility (6 cases).

The control subjects (n = 14) are men members of infertile couples undergoing an *in vitro* fertilization cycle during the same week as the case-subjects and having normal semen parameters and a fertilization rate ≥ 65%. The causes of infertility in this group were: tubal infertility (6 cases), polycystic ovary syndrome (1 case), endometriosis (5 cases) and unexplained infertility (2 cases).

All the female partners of both the case and the control subjects must have at least 3 mature oocytes inseminated with husband’s fresh ejaculated spermatozoa. These patients underwent pituitary down-regulation using a GnRH analog (Decapeptyl 0.1 mg subcutaneously per day, Ipsen, France) starting on the 21st day of the previous cycle and continuing until the day of hCG administration. When E2 levels fell below 40 pg/ml, patients underwent stimulation with FSH (Gonal F, Merck Serono, France). Follicular development was monitored by E2 levels and transvaginal ultrasound measurements daily, beginning 5 to 6 days of stimulation. An injection of hCG (Ovitrelle, Merck Serono, France) was given when at least 3 leading follicles reached a mean diameter ≥17 mm and the serum E2 level exceeded 250 pg/ml per follicle. Transvaginal follicular aspiration was performed under ultrasound guidance 36 hours after the administration of hCG.

### Samples

Informed consent was obtained from all subjects for using their semen and blood samples in this study. The study was approved by the local Ethical CPP (Comité de Protection des Personnes).

Samples were collected in the IVF laboratory of Tenon Hospital. Semen samples were obtained at day 1 after sperm insemination (when assessing oocytes’fertilization) or later by calling the patients for a new semen sample.

At day 1 after sperm insemination, for each patient, the entire remained semen sample and the pellet obtained after density gradient are pooled so that we collect the maximum of spermatozoa to have a sufficient protein extract later. Then, spermatozoa were separated from seminal plasma by centrifugation (10 min, 12000 rpm). The supernatant is removed and 1 ml PBS (Phosphate Buffer Solution) is added to the pellet. A second centrifugation is carried, and just after, the supernatant is removed and the final pellet is frozen at −80°C.

If a semen sample is not obtained at day one after IVF, the patient is asked for a new semen sample after a sexual abstinence of 3 to 5 days. After a spermatozoa count, the semen is centrifuged, the supernatant removed and the pellet frozen at −80°C.

Centrifugation and supernatant removal allow us to eliminate sACE normally present in the seminal plasma.

### Protein extraction

Frozen pellets of spermatozoa were thawed, resuspended in cold PBS and centrifuged twice (5 min, 12000 rpm). The supernatant was removed and spermatozoa were extracted by mixing the last pellet with 500 μl RIPA1X buffer and grinding them. After a last centrifugation, the supernatant was removed and stored at −80°C. The supernatants containing ACE were quantified by Biorad Protein Assay (Biorad, France).

### Gel electrophoresis and protein blotting

Ten to 20 μg of total proteins were subjected to 7.5% SDS-PAGE (Biorad, France), and blotted onto polyvinylidene difluoride membrane (Biorad, France). A prestained protein ladder (BenMark Pre-stained Protein Ladder, Invitrogen, France) was used to determine the molecular weight of the protein. So together with anti-ACE antibody specificity and molecular weight (100 kDa) we are sure that it is the testicular isoform, the somatic one is 170 kDa and is absent or too weak in the protein extracts. After blocking with 5% dried milk in PBS-T (0.05% Tween 20 in PBS), the membrane was incubated with a diluted (1/100th) goat anti ACE polyclonal antibody (ACE C20 peptide, Sc-12187, TEBU-BIO SA, France) at 4°C overnight. This antibody have been previously tested for its cross reactivity by using the 293 T whole cell lysate as a positive control (Santa Cruz Biotechnology, France) and the sc-12187 Blocker Peptide (Santa Cruz Biotechnology) as a negative one. The membrane was washed three times (10 min for each) in PBS-T, and incubated with rabbit anti-goat (1/10000th) (Rabbit anti-goat IgG HRP conjugate, Sc-2768, TEBU-BIO SA, France) for 1 hour at room temperature. After washing 3 times in PBS-T, the immunoreactive signal was detected by using ECL™ Western Blotting Detection Reagents (Amersham, France). These membranes were then incubated in a tris-SDS-β-mercaptoethanol (30 min in a wet chamber at 50°C). After blocking as mentioned above, the membrane was incubated with a diluted goat (1/500th) anti Pyruvate Kinase (standard protein) polyclonal antibody (Biogenesis, France) overnight at 4°C. Incubation with the secondary antibody (the same as the one used for ACE) and detection of the immunoreactive signal were done as mentioned above.

### DNA extraction and PCR

A total of 10 case-subjects and 14 control-subjects were screened for PCR of the tACE gene. Genomic DNA was extracted from peripheral blood using QIAamp Blood Kit (Qiagen, France). Specific primers were designed to amplify each of the 14 exons of the tACE. The primer sequences are available upon request.

The PCR amplification was carried out in a total volume of 30 μl reaction mixture containing MgCl2 (1.5 mM), 0.1 mM of each dNTP, 100 ng of genomic DNA, 0.11 μm of each primer (primers are previously diluted at a 3.3 μM concentration) and 1 IU of gold Taq DNA polymerase. We used a touch-down PCR. The cycling profile consisted of denaturation at 95°C for 30 seconds, annealing at 55°C for 30 seconds, and extension at 72°C for 30 seconds except for the first cycle where denaturation was extended to 9 minutes.

### Direct DNA sequencing

Purified PCR products were submitted to direct sequencing. Sequencing reaction were performed in forward and reverse orientations using the ABI BigDye Terminator v1.1 cycle sequencing kit (Applied Biosystems, France).

Statistical analysis was performed using chi-squared test as a test of significance. A p ≤ 0.05 was considered as significative.

## Results

The mean age of the case-subjects was 41.4 ± 7.5 years and the mean age of their female partners was 34.5 ± 4.5 years at the time of the screening. The mean age of the control-subjects and their female partners was respectively 40.8 ± 3.9 and 33.8 ± 3.75 years. The average number of oocytes retrieved was 9.44 ± 3.32. The average number of mature oocytes was 7.65 ± 4.7.

### Characterization of tACE in the spermatozoa pellets

A total of 10 cases and 14 control-subjects’ samples were extracted and western blotted.

**Figure 1:**
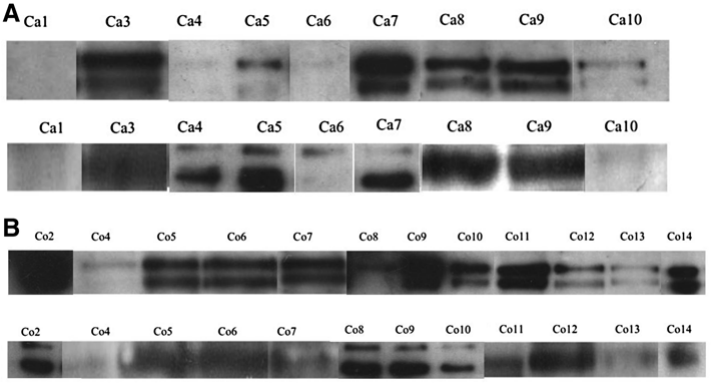
**Assessment of tACE in spermatozoa pellets by Western-Blot. A.** Assessment of tACE in spermatozoa pellets of the case-subjects by Western-Blot. Lane 1: tACE: Goat anti-ACE polyclonal antibody(1/100^ème^), rabbit anti-goat antibody (1/10000^ème^). Lane 2: Pyruvate Kinase: Pyruvate kinase polyclonal antibody (1/500^ème^), rabbit anti-goat antibody (1/10000^ème^). **B.** Assessment of tACE in spermatozoa pellets of the control-subjects by Western-Blot. Lane 1: tACE: Goat anti-ACE polyclonal antibody(1/100^ème^), rabbit anti-goat antibody (1/10000^ème^). Lane 2: Pyruvate Kinase: Pyruvate kinase polyclonal antibody (1/500^ème^), rabbit anti-goat antibody (1/10000^ème^). Co: Control-subjects. Ca: Case-subjects.

As shown in Figure 1, the two lanes correspond to the glycosylated and non-glycosylated forms of the tACE. This protein is approximately 100 kDa.

Three case-subjects (4, 5 and 6) had no tACE expression when the lanes were compared to the standard protein (pyruvate kinase lanes). However, one control-subject has also no tACE expression. There’s no significant difference between the case and the control-subjects. The power of the statistical analysis is 34% (α = 0.05).

### Sequencing of the tACE gene exons

Genomic DNA of all subjects (10 case-subjects and 14 control-subjects) was used for PCR amplification. No mutation was present in tACE gene exons from all these subjects.

### Results of intra cytoplasmic sperm injection (ICSI) after IVF failure in case patients

All the case subjects having an IVF failure underwent an ICSI cycle. Results are shown in Table 1.

**Table 1:** **Outcome of ICSI after IVF failure in case-subjects**

Case subjects	Survival rate of spermatozoa 24 hours after IVF	Number of spermatozoa bounded to the ZP 24 hours after IVF	tACE expression	ICSI rank	Fertilization rate in ICSI	ICSI results
1	40–60%	5	Too weak for interpretation	1	82%	Biochemical pregnancy
2	>60%	0	+	1	89%	Pregnancy loss
				2	92%	Baby: girl
				3	34%	Implantation failure
3	>60%	1 à 5	+	1	100%	Implantation failure
				2	66%	Implantation failure
				3	50%	Biochemical pregnancy
				4	0%	Fertilization failure
4	>60%	2 à 4	−	1	57%	Baby boy
5	>60%	0 à 5	−	1	100%	Baby boy
6	40–60%	2 à 5	−	1	77%	Baby boy
7	>60%	0 à 10	+	1	87%	Baby boy
8	>60%	1 à 3	+	1	0%	Fertilization failure
9	40–60%	0	+	1	75%	Baby girl
10	>60%	5	+	1	43%	Implantation failure

All the case subjects underwent at least one ICSI cycle. Among them, 90% have normal fertilization rate (34 to 100%) and 70% of them have excellent fertilization rate (≥65%). The total clinical pregnancy rate per cycle is 47%. Only two patients have a total fertilization failure when undergoing ICSI, despite a sufficient number of mature oocytes (8 mature oocytes for the case-subject n°3 and 10 mature oocytes for the case subject n°8). These non-fertilized eggs were fixed and examined after Giemsa staining. Only 3 mature oocytes for patient n°3 and 2 oocytes for patient n°8 could be interpreted. They all had a premature condensation of the sperm chromatin (PCC). This condition is due to a cytoplasmic immaturity of the eggs and leads to a lack of oocyte activation [19].

## Discussion

In the present study, western-blot was performed on 10 case-subjects and 14 control-subjects. Three case-subjects and one control-subject had no tACE expression. There was no statistic difference between the two groups. The power of the statistical analysis is 34% (α = 0.05).

PCR was performed on 12 blood samples; no mutation was detected in the tACE DNA sequences.

When we observed the phenotypes of case-subjects (spermatozoa survival and number of spermatozoa bound to the ZP), we did not find any significant difference between case subjects without tACE expression and those who have normal tACE expression (Table 1).

We underwent this study in the optics to find a cause to certain cases of male infertility presenting a phenotype similar to that of tACE gene knockout mice models.

Many previous studies using tACE knockout mice provided further information about the function of tACE in male fertility. Krege et al. [15] demonstrated that homozygous ACE knockout male mice sire few pups despite mating normally whereas homozygous female mutants are fertile. Hagaman et al. [16] observed that expression of ACE in sperm is important for egg fertilization. Sperm lacking tACE are deficient in transport within the oviduct and in binding to ZP [16]. It is on these results that we established the criteria of selection of our case-subjects.

However, in the study of Hagaman et al. [16], the average number of spermatozoa of male homozygous ACE−/− mice counted in wild female mice oviducts one hour after mating was sharply lower than that of wild male mice counted in the same conditions [16]. Consequently, we can suppose that in our study, the inclusive criteria of our case subjects were too drastic. We should have included men with IVF failure and either a low semen survival rate (i.e: low motility post-capacitation *in vitro*) or a decrease of the percentage of rapid progressive motility (type a) [18] although relationship between this motility defect and the tACE seems difficult to establish. Indeed, only Shibahara et al. [20] found a negative correlation between the membrane tACE activity and semen motility. Because of our drastic inclusive criteria, only 10 case-subjects were included in this study and we did not find any significant difference. Moreover, the power of the statistical analysis was too low (34%) and is certainly due to the low number of the patients included. We should have between 45 and 50 case-patients included and the same number in the control-group with a power of 80% to have a significant difference.

ICSI overcame this condition of total fertilization failure for 8 case subjects. However, two patients have a total fertilization failure when undergoing ICSI, despite a sufficient number of mature oocytes. Only 3 mature oocytes for patient n°3 and 2 oocytes for patient n°8 could be interpreted. They all had a premature condensation of the sperm chromatin (PCC). This condition is due to a cytoplasmic immaturity of the eggs and leads to a lack of oocytes activation [19]. Thus, despite our drastic criteria, two couples have both a female and male factors as causes of total fertilization failure.

The implication of the tACE in male fertility is far from having clearly been clarified. Métayer et al. [21] found that ejaculated mouse sperm have no remaining ACE. Thus, the sperm physiological changes may not relate to a direct role of ACE in capacitation, acrosomal reaction, or sperm-oocyte interaction. They have demonstrated in the rat and mouse that the germ cell membrane-bound ACE is completely released under an active form as the sperm passed through the proximal caput of the epididymis. Nevertheless, Yamaguchi et al. [22] showed that tACE is localized exclusively on the head of sperm from the cauda epididymis suggesting that tACE is involved in sperm-ZP interaction. Some authors reported that GPIase activity of ACE is necessary for sperm to acquire ZP binding activity [23]. Kondoh et al. postulated that some GPI (Glycosyl Phosphatidyl Inositol) anchored proteins on the sperm surface might play a role in ZP interaction after cleavage or that tACE may expose a ZP binding factor by shedding GPI-anchored proteins that block binding [23]. However, Fuchs et al. [24] indicated that sperm ACE is unable to cleave GPI anchored proteins but plays its crucial role in binding to the ZP thanks to its dicarboxypeptidase activity.

Testicular ACE can thus play a physiological role in oocyte fertilization, but at which step? Motility post-capacitation explaining the difficulty of ascension in the female oviducts of spermatozoa ACE−/−? Spermatozoa-ZP interaction? Spermatozoa binding to the oelemma? The most constantly described abnormality is the defect of spermatozoa-ZP interaction.

According to our literature analysis, tACE plays a crucial role in fertilization in rodents as demonstrated by tACE knock-out mice models. We based our study on these rodent models to look for a lack of the expression of the tACE in the semen of patients carrying the same phenotype: a failure of fertilization during an IVF cycle with no spermatozoa bound to the ZP despite normal semen parameters. In western-blot, we did not find any significant difference between the case and the control patients. The PCR was performed on blood samples of 10 case-subjects and 14 control-ones. No mutation was detected in the tACE DNA sequences. It would be advisable for our study to enlarge the criteria of selection to include all the animal model phenotypes, particularly men with IVF failure and either a low semen survival rate (i.e: low motility post-capacitation) or a decrease of the percentage of rapid progressive motility (type a) [18]. Besides, a multicentric study will allow us to include a sufficient number of subjects to evidence a significantly statistical difference between the case and the control subjects. Other techniques should be applied such as proteomic mapping, spectrophotometry or micro-array. ICSI offers a particularly effective therapeutic solution to these cases of male infertility but does not tackle the mechanisms in cause.

## Abbreviations

ACE: Angiotensin converting enzyme
tACE: Testicular angiotensin converting enzyme
sACE: Somatic angiotensin Converting Enzyme
ZP: Zona pellucida
WHO: World Health Organization
IVF: *In vitro* fertilization
ICSI: Intra Cytoplasmic sperm injection
RAS: Renin angiotensin system
CPP: Comitéde protection des personnes
PCC: Premature chromatin condensation
GPI: Glycosyl phosphatidyl inositol
